# Influence of light absorption rate on the astaxanthin production by the microalga *Haematococcus pluvialis* during nitrogen starvation

**DOI:** 10.1186/s40643-023-00700-0

**Published:** 2023-11-09

**Authors:** Khadija Samhat, Antoinette Kazbar, Hosni Takache, Ali Ismail, Jeremy Pruvost

**Affiliations:** 1 Oniris, CNRS, GEPEA, UMR 6144, Nantes University, 44600 Saint-Nazaire, France; 2 Platform for Research and Analysis in Environmental Sciences, Doctoral School of Science and Technology, Lebanese University, Rafic Hariri Campus, Beirut, Lebanon; 3 Algosource, 7 Rue Eugène Cornet, 44600 Saint-Nazaire, France; 4 Bioprocess Engineering, Wageningen University and Research, Wageningen, Netherlands; 5 Bio-Information Research Laboratory (BIRL), The Higher Institute of Biotechnologies of Paris (Sup’biotech), 66 Rue Guy Môquet, 94800 Villejuif, France

**Keywords:** *Haematococcus pluvialis*, Photobioreactor, Photosynthesis, Light transfer, Astaxanthin accumulation, Nitrogen starvation

## Abstract

Large amounts of astaxanthin (about 4% DW) can be produced under nitrogen starvation of *Haematococcus pluvialis* in photobioreactors (PBRs) exposed to high light conditions to induce a light stress. However, in PBR, the large biomass concentration usually achieved leads to strong light attenuation conditions, which makes complex the analysis of this “light stress”. This study aims to elucidate the role of light transfer in astaxanthin cell content and productivity from the microalga *Haematococcus pluvialis* during nitrogen starvation. *Haematococcus pluvialis* was cultivated in a flat-panel PBR in a batch mode with sudden nitrogen starvation conditions and an incident photon flux density (PFD) of 250 µmol_hν_ m^−2^ s^−1^. Different initial biomass concentrations (${C}_{{x}_{0}}$) were evaluated, 0.21, 0.52, 1.39 and 2.21 kg m^−3^. As a result, spectral mass absorption cross-sections of *Haematococcus pluvialis* were measured at different times during nitrogen starvation, and were used to relate the mean rate of photon absorption (MRPA) to the astaxanthin productivity. A minimum initial MRPA of 7000 ± 500 µmol_hν_ kg_x_^−1^ s^−1^ was found necessary to trigger large accumulation of astaxanthin in *Haematococcus pluvialis* cells (up to 3.21% DW) during nitrogen starvation conditions. The results also demonstrated the link between the MRPA and the daily astaxanthin productivity of *Haematococcus pluvialis* cultures, introducing then the MRPA as a physical quantity of interest for a rational optimization of the light culture conditions in PBRs.

**Graphical Abstract:**

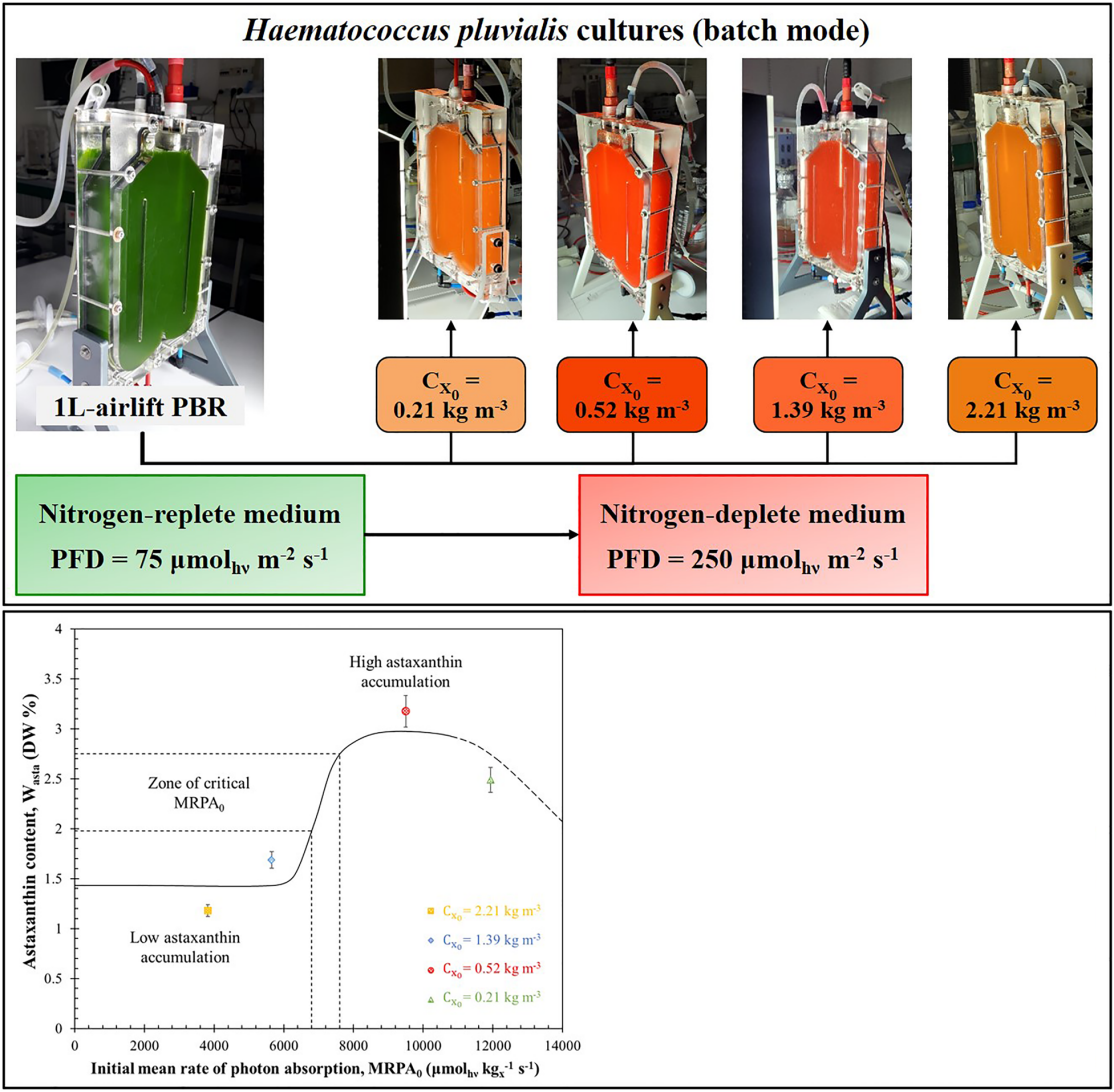

**Supplementary Information:**

The online version contains supplementary material available at 10.1186/s40643-023-00700-0.

## Introduction

Astaxanthin (3,3′-dihydroxy-β,β-1-carotene-4,4′-dione) is one of naturally occurring xanthophylls, which, together with carotenes, constitute a class of more than 600 molecules called carotenoids. It is a biologically active material that is useful in many applications, including nutrition supplement, pharmaceuticals, animal feedstock, and cosmetics. It also exhibits higher antioxidant activity than other carotenoids like β-carotene and vitamin E (Guerin et al. 2003).

The freshwater unicellular microalga *Haematococcus pluvialis* has been recognized as the best source of natural astaxanthin due to its potential to accumulate large amounts of astaxanthin (Lorenz and Cysewski 2000; Ranjbar et al. 2008). Most companies employ a two-step process to cultivate *H. pluvialis* for astaxanthin production (Harker et al. 1996; Olaizola 2000; Fábregas et al. 2001; Han et al. 2013; Liu et al. 2020). This process has been developed based on the life cycle and cell biology of *H. pluvialis*, which mainly consists of at least two stages, the green stage when replete-nutrient medium is supplied under favorable growth conditions, and the red stage when nutrients (usually nitrogen) are depleted under conditions of stress. During the green stage, the cells can reproduce and accumulate biomass but not astaxanthin, while during the red stage, the cells lose the ability of division and mobility but are capable of accumulating as high as 5% of astaxanthin of dry biomass weight (Kobayashi et al. 1997; Fábregas et al. 2001, 2003; Kang et al. 2005).

Light and nitrogen are the two most effective factors that influence growth and astaxanthin synthesis in *H. pluvialis*. Nitrogen starvation combined with high light is known as the best way to trigger astaxanthin accumulation in *H. pluvialis* (Boussiba 2000; Scibilia et al. 2015; Zhang et al. 2018; Rizzo et al. 2022). It was widely studied for many years, especially for enhancing the production of *H. pluvialis* biomass enriched in astaxanthin. One of the consequences of nitrogen starvation is the reduction in the chlorophyll concentration in the cells which greatly modify the light absorption of microorganisms, thus affecting their ability to accumulate astaxanthin (Solovchenko et al. 2011).

Along with nitrogen starvation, the term light stress has often been used in the literature as a necessary condition for large astaxanthin productivity (Hu et al. 2008). However, the concept of light stress has remained qualitative, especially when considering the complexity of its definition in algal culture system. Indeed, light penetration in culture volume depends on the concentration of *H. pluvialis* suspension as well as pigment content (i.e., radiative properties of the cells) (Lee et al. 1987). Therefore, optimizing light stress for optimized astaxanthin production involves to relate all these effects to accurately quantify the light stress. For example, an appropriate light stress will induce an astaxanthin over accumulation, which in turn will modify light penetration in the culture volume, and then the light stress applied to cells.

The objective of this study is to explore the potential of utilizing a physical parameter, the mean rate of photon absorption (MRPA), which provides information on the rate of light energy uptake by photosynthetic organisms. This study aims to contribute to the understanding of processes related to light energy and their implications for astaxanthin production. The relation between light absorption rate and astaxanthin production rate by the microalga *H. pluvialis* grown in nitrogen starvation condition under high light intensity in a flat-panel PBR is then investigated. For this purpose, four biomass cultivations were performed in batch mode and continuous light intensity of 250 µmol_hν_ m^−2^ s^−1^. Different initial biomass concentrations ${C}_{{x}_{0}}$ of 0.21, 0.52, 1.39 and 2.21 kg m^−3^ were applied leading to several light attenuation conditions. Results were then analyzed in terms of growth kinetics and astaxanthin accumulation, and also of light transfer conditions as represented by the mean rate of photon absorption MRPA. In the same way as Kandilian et al. (2014) showed that the MRPA controls the accumulation of intracellular reserves by numerous microalgae as triacylglycerol, it was expected here similar results on astaxanthin accumulation from *H. pluvialis*. The results would be instrumental in optimizing and defining protocols for astaxanthin production in PBRs.

## Theoretical considerations

### Light transfer modeling

Because of the light attenuation that occurs in the culture volume, numerous kinetic models have been developed for coupling microalgae biomass and/or metabolite productivity to light transfer in the culture volume (Cornet et al. 1992; Grima et al. 1994; Takache et al. 2009, 2012; Cornet and Dussap 2009; Béchet et al. 2013). The specific local rate of photon absorption (LRPA), noted $\mathcal{A}$ (Cassano et al. 1995), revealed of interest when coupling light-related kinetics (i.e., photosynthesis) to light transfer conditions. $\mathcal{A}$ represents the amount of photon absorbed in the spectral range of photosynthetic active radiation (PAR, between 400 and 700 nm) per unit weight of biomass and per unit time, and is expressed in µmol_hν_ kg_x_^−1^ s^−1^. $\mathcal{A}$ depends on the absorption cross-section ${A}_{abs,\lambda }$ of the microalgae (and then of cells pigmentation) and of the spectral fluence rate ${G}_{\lambda }\left(z\right)$ obtained at a given depth of culture. As a result, ${\mathcal{A}}_{\lambda }\left(z\right)$ is a local value ${{(\mathcal{A}}_{\lambda }\left(z\right)=A}_{abs,\lambda }{G}_{\lambda }\left(z\right))$ which has to be averaged on both the PAR region and the total culture volume to obtain the mean rate of photon absorption (MRPA or < >$\mathcal{A}$).

For a flat-panel PBR, as used in this study, it is simply obtained by averaging local values $\mathcal{A}$ over the culture depth *z* (Pruvost and Cornet 2012):

1 $$MRPA = \left\langle {\mathcal{A}} \right\rangle \, = \,\frac{1}{L}\,\int_{400}^{700} {\int_{0}^{L} {{\mathcal{A}}_{\lambda } \left( z \right)dzd\lambda = \frac{1}{L}} } \,\int_{400}^{700} {\int_{0}^{L} {A_{abs,\lambda } G_{\lambda } \left( z \right)dzd\lambda } } ,$$

where ${A}_{abs,\lambda }$ is the spectral mass absorption cross-section of the microalgae (in m^2^ kg^−1^), ${G}_{\lambda }\left(z\right)$ is the local fluence rate at depth *z* (in µmol_hν_ m^−2^ s^−1^), *L* is the thickness of the PBR (in m) and λ is the light wavelength (in nm).

In the case of microalgal culture, the local spectral fluence rate ${G}_{\lambda }\left(z\right)$ can be obtained by solving the radiative transfer equation (Jonasz and Fournier 2007). For geometries responding to the one-dimensional hypothesis, like in the case of flat-panel PBR, the two-flux model proved also effective to properly describe light diffusion and absorption phenomena in algal suspensions (Pottier et al. 2005). This requests however to determine the backward scattering ratio defined as the fraction of the radiation scattered backwards, which is estimated from the suspension’s scattering phase function. It was measured for different species of microalgae (Kandilian et al. 2014, 2019), but for *H. pluvialis* this value has not yet been measured. But because the backward scattering ratio is very low for microalgae cells, Lee et al. (2014) and Kandilian et al. (2016) demonstrated that a simplified method only based on absorption properties could be used to determine the fluence rate field and then MRPA with negligible error, leading to:

2 $$G_{\lambda } \left( z \right) = q_{\lambda ,0} \exp \left( { - A_{abs,\lambda } C_{x} z} \right) + \rho_{\lambda } q_{\lambda ,0} \exp \left( { - A_{abs,\lambda } C_{x} \left( {2L - z} \right)} \right),$$

where *q*_*λ,0*_ is the incident PFD (in µmol_hν_ m^−2^ s^−1^) and *ρ*_*λ*_ represents the diffuse reflectance of the PBR back wall (*ρ*_*λ*_ = 0 for transparent back wall).

Following Eq. 2, the simplified expression for the fluence rate ${G}_{\lambda }\left(z\right)$ depends only on the spectral mass absorption cross-section ${A}_{abs,\lambda }$ of the microalgal suspension, which has to be accurately determined using a spectrophotometer with an integrating sphere to take into account from the light scattering by microalgae cells (Kandilian et al. 2016). This method was recently used and validated by Ferrel Ballestas et al. (2023) for a culture of *Chlamydomonas reinhardtii* cultivated in PBRs under progressive nitrogen starvation conditions.

The PAR-averaged fluence rate $G\left(z\right)$ can be then obtained by integrating the local values ${G}_{\lambda }\left(z\right)$ over the PAR region as:

3 $$G\left( z \right) = \mathop \smallint \limits_{400}^{700} G_{\lambda } \left( z \right)d\lambda .$$

## Materials and methods

### Strain and culture medium

The algal strain *Haematococcus pluvialis* SAG 34–7 was obtained from the Culture Collection of Algae at the University of Göttingen, Germany. It was cultivated in modified Bold’s Basal Medium (BBM; (Nichols and Bold 1965)) with the following composition (in mM): NaNO_3_ 8.824, MgSO_4_⋅7H_2_O 0.913, CaCl_2_⋅2H_2_O 0.17, Na_2_EDTA⋅2H_2_O 0.134, FeSO_4_⋅7H_2_O 0.05, K_2_HPO_4_ 0.861, KH_2_PO_4_ 0.9, ZnSO_4_⋅7H_2_O 7.72 × 10^−4^, Co(NO_3_)_2_⋅6H_2_O 1.51 × 10^−4^, CuSO_4_ 6.25 × 10^−7^, H_3_BO_3_ 4.6 × 10^−5^, MnCl_2_⋅4H_2_O 9.15 × 10^−6^, Na_2_MoO_4_ 1.063 × 10^−3^ and NaHCO_3_ 15.

For nitrogen starvation experiments, the nitrate (NaNO_3_) was omitted from the medium while maintaining the other components constant.

### Cultivation method

All experiments were performed in a 1-L airlift-type flat-panel PBR (thickness *L* = 3 cm). The PBR was described in more detail in Pruvost et al. (2009).

The PBR was continuously illuminated on one side by a white LED light panel with adjustable PFD. The PBR was set with a complete loop of common sensors and automation for microalga culture, namely pH, temperature, and gas injections (CO_2_ and air). The pH was measured using a pH sensor (InPro48XX, Mettler-Toledo AG, Greifensee, Switzerland) and was set to 7.5 ± 0.5 by the injection of CO_2_ inside the PBR when the measured pH exceeded the set point, and the temperature was kept stable at room temperature in a temperature-controlled room set at 21 ± 1 °C. The incident PFD was measured over the PAR region at nine different locations on the inside surface of the PBR using a quantum light sensor (Li-250A, Li-COR, Lincoln, NE). The PBR was sterilized for 30 min using a 5 mM peroxyacetic acid solution and rinsed twice with sterile deionized water, before starting each experiment.

Initially, the PBR was inoculated with batch-grown green *H. pluvialis* cells at optimal growth condition (i.e., low PFD of 75 µmol_hν_ m^−2^ s^−1^). Then the culture was collected to inoculate the nitrogen starvation experiments and thus trigger carotenoids accumulation.

A specific volume of green *H. pluvialis* culture was harvested and centrifuged at 10000 g (ThermoScientific Sorvall RC 6 Plus, Massachusetts, USA) for 10 min at 4°C, washed twice with nitrogen-free BBM medium and injected into the new airlift-PBR filled with the nitrogen-free medium and exposed to a continuous incident PFD of 250 µmol_hν_ m^−2^ s^−1^. The volume of culture was chosen based on the desired initial biomass concentration ${C}_{{x}_{0}}$ of each sudden nitrogen starvation experiment, following the protocol set by Van Vooren et al. (2012) for lipids accumulation. The growth kinetics of each culture under nitrogen starvation conditions as well as that of the production of astaxanthin were studied during 10 days of starvation in the PBRs in batch mode.

### Analytical methods

#### Dry weight biomass concentration

Microalgae dry weight concentration *C*_*x*_ was measured gravimetrically by filtering given culture volume through a pre-dried and pre-weighed glass-fiber filter (Whatman GF/F, 0.47 μm pore size, VWR, France). The filters were dried at 105 °C for at least 24 h and then reweighed after being cooled in a desiccator for 10–15 min. The samples were analyzed in triplicates and the reported biomass concentration corresponded to the mean value.

#### Morphological observation

The morphological changes in cells were observed using an optical microscope (Zeiss, Axio, Imager.M2m, Jena, Germany) connected to a CCD camera (Zeiss, AxioCam MRc, Jena, Germany).

#### Pigment concentration

Pigments were extracted and quantified spectrophotometrically. According to Qiu et al. (2018), a volume V_1_ (in mL) of *H. pluvialis* culture was first centrifuged at 13400 rpm for 15 min. The medium was discarded and the cells were resuspended in a volume V (in mL) of dimethyl sulfoxide (DMSO) and then incubated at 50 °C for 1–2 h in the dark until the sample turned white. After cooling, the pigment extract was diluted with a volume V_2_ (in mL) of 90% acetone (the ratio of DMSO:90% acetone should be equal to 1:4) and was then centrifuged at 13400 rpm for 15 min. The optical density OD_λ_ of the supernatant was measured at 630, 645, 665 and 750 nm with a UV–vis spectrophotometer (JASCO V-630, France). All extractions were performed in triplicates. Chlorophyll a (*chl-a*) and b (*chl-b*) concentrations (in g m^−3^) were calculated as follows (Strickland et al. 1968):

$$C_{chl - a} = \, [11.6 \, (OD_{665} \, - \, OD_{750} ) 1.31 \, (OD_{645} \, - \,OD_{750} ) 0.14 \, (OD_{630} \, - \,OD_{750} )]\,\frac{{v_{2} }}{{v_{1} l}},$$

4 $$C_{chl - b} = \, [20.7 \, (OD_{645} OD_{750} ) 4.34 \, (OD_{665} OD_{750} ) 4.42 \, (OD_{630} OD_{750} )]\,\frac{{v_{2} }}{{v_{1} l}},$$

where *V*_*1*_ is the culture sample volume (in mL), *V*_*2*_ is the volume of added acetone (in mL) and *l* is the cuvette path length (1 cm).

The corresponding mass fraction of pigment ‘*i*’ per dry weight of biomass *W*_*i*_ (in DW %) can be estimated as:

5 $$W_{i} \, = \,\frac{{c_{i} }}{{c_{x} }} \,100,$$

where *C*_*i*_ is the pigment concentration (in g m^−3^).

#### Astaxanthin content

Astaxanthin was extracted in DMSO and quantified spectrophotometrically according to the method published by Boussiba et al. (1992). The harvested algae cells V_1_ were collected by centrifuging at 11000 rpm for 10 min, first treated to destroy the chlorophyll with a solution of 5% (*w*/*v*) KOH in 30% (*w*/*w*) methanol at 70 °C for 10 min. The supernatant was discarded, and the remaining pellet was extracted with a volume V_2_ of DMSO after adding 100 µL of acetic acid and applying homogenization (by vortex) for astaxanthin recovery (acetic acid should be added before the addition of the DMSO otherwise the pellet will agglomerate and it will be difficult to perform the extraction). The extract was then heated for 30 min at 70 °C. The red supernatant was collected (after centrifugation for 10 min at 11000 rpm), the optical density at 490 nm was measured and the astaxanthin concentration *C*_*asta*_ (in g m^−3^) was calculated according to the following equation (Zhang et al. 2018):

6 $$C_{asta} = \, 4.5 \, OD_{490} V_{1} \,V_{2} ,$$

where *V*_*1*_ is the culture sample volume (in mL), *V*_*2*_ is the volume of added DMSO (in mL) and *OD*_*490*_ is the extract optical density at 490 nm.

The astaxanthin content *W*_*asta*_ (in DW %) was then calculated:

7 $$W_{asta} = \,\frac{{c_{asta} }}{{c_{x} }}\,100.$$

#### Experimental measurements of radiation characteristics

The radiation characteristics (i.e., the mass absorption cross-sections ${A}_{abs,\lambda }$) of the microorganisms were measured using the method described by Kandilian et al. (2016). The normal-hemispherical transmittance ${T}_{nh,\lambda }$ and reflectance ${R}_{nh,\lambda }$ of *H. pluvialis* suspensions, with biomass concentration ranging from 0.1 to 10 kg m^−3^, were measured using the internal integrating sphere accessory (Agilent Cary DRA-2500, Santa Clara, CA) of the UV–Vis–NIR spectrophotometer (Agilent Cary 5000, Santa Clara, CA).

The microalgal samples were centrifuged at 11000 rpm for 15 min at 15°C and washed twice with phosphate buffer saline (PBS) solution before measurements, in order to avoid absorption and scattering by the growth medium, and then suspended in PBS. The volume of culture sampled and PBS used were chosen based on the biomass concentration desired for optical measurements (0.1–10 kg m^−3^). Quartz cuvettes of 1 cm depth were used (110-10-40 Hellma Analytics, Müllheim, Germany). The ${T}_{nh,\lambda }$ and ${R}_{nh,\lambda }$ experimentally measured in the wavelength range from 350 to 750 nm (1 nm spectral resolution) were then used as input parameters in the inverse method to retrieve the spectral mass absorption cross-sections ${A}_{abs,\lambda }$ of *H. pluvialis*. The ${A}_{abs,\lambda }$ were then used to calculate the local fluence rate ${G}_{\lambda }\left(z\right)$ and the MRPA < $\mathcal{A}$>.

Note that in batch cultivation, the mass absorption cross-sections ${A}_{abs,\lambda }$ of the microalgae as well as the biomass concentration *C*_*x*_ are all time-dependent. In practice, with daily sampling of the culture, the daily average areal astaxanthin productivity ${\overline{S} }_{asta}$ and the daily average mean rate of photon absorption < $\overline{\mathcal{A} }$> of a batch culture were calculated as:

8 $$\overline{S}_{asta} \left( {t_{i} } \right) = \frac{{C_{asta} \left( {t_{i} } \right) - C_{asta} (t_{i} - 1)}}{{t_{i} - t_{i - 1} }} L,$$

9 $$\left\langle {\overline{{\mathcal{A}}} } \right\rangle \,\left( {t_{i} } \right) = \frac{{MRPA_{avg} \left( {t_{i} } \right) + MRPA_{avg} (t_{i} - 1)}}{2},$$

where* t*_*i*_ and *t*_*i-1*_ correspond to two consecutive sampling times 1 day apart.

## Results and discussion

Four sudden starvation experiments were performed with different initial biomass concentrations ${C}_{{x}_{0}}$ of 0.21, 0.52, 1.39 and 2.21 kg m^−3^. In all cases, the front face of the PBR was exposed to an incident PFD of 250 µmol_hν_ m^−2^ s^−1^.

### Effect on biomass growth

**Fig. 1:**
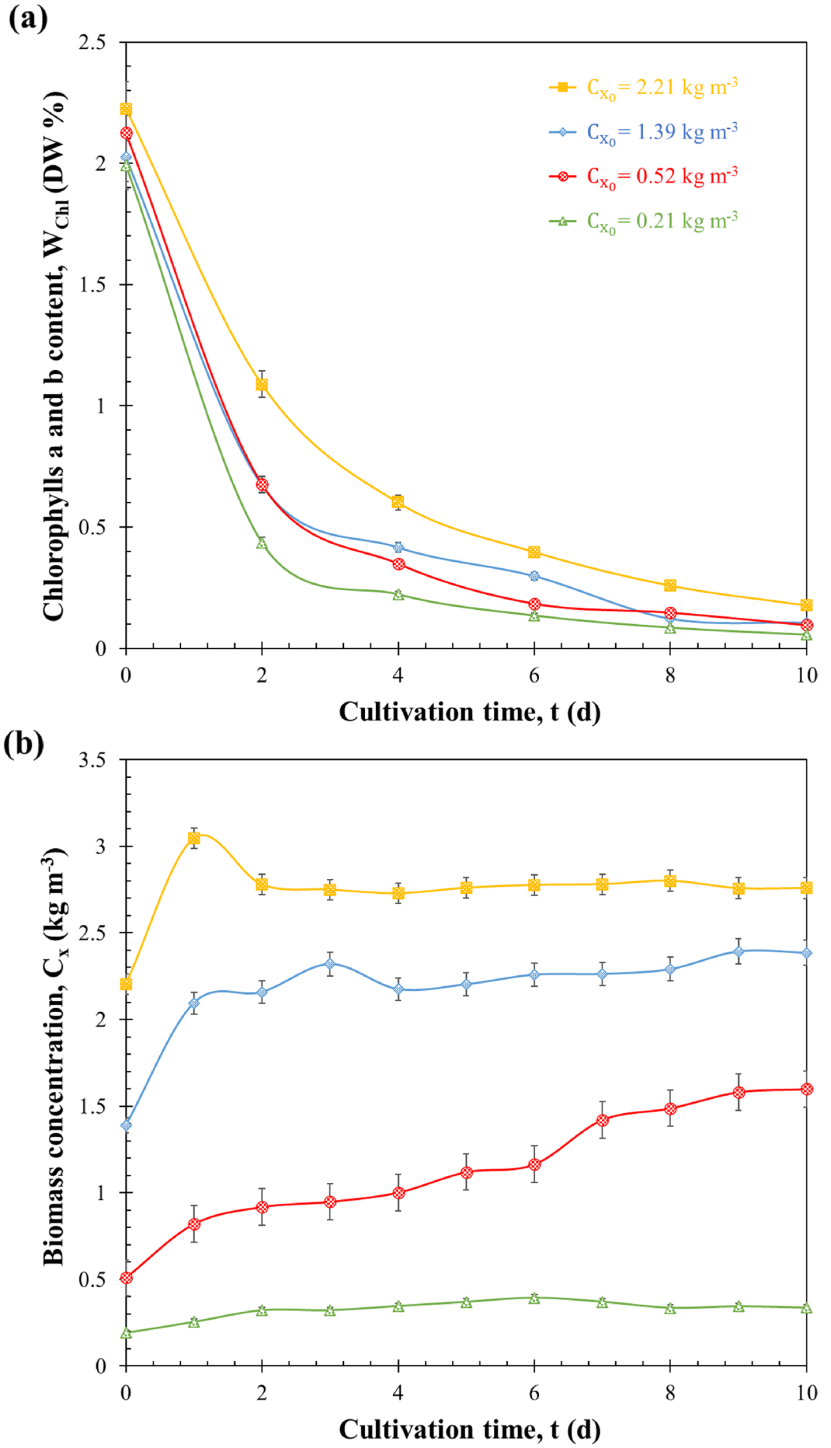
Chlorophyll content W_chl_ (**a**) and biomass concentration C_x_ (**b**) of H. pluvialis during sudden nitrogen starvation of batch cultures exposed to PFD of 250 µmol_hν_ m^−2^ s^−1^ with initial biomass concentrations ${\mathrm{C}}_{{\mathrm{x}}_{0}}$ equals to 0.21, 0.52, 1.39 and 2.21 kg m^−3^ as a function of cultivation time t. Data shown as mean ± SD, *n* = 3

The growth of *H. pluvialis* under sudden nitrogen starvation was characterized by following the temporal evolution of the biomass concentration *C*_*x*_ and chlorophyll a and b content *W*_*chl*_. The results are presented in Fig. 1.

For all nitrogen starvation experiments, Fig. 1a clearly shows a decrease in chlorophyll content *W*_*chl*_ with starvation. This drastic decrease was observed immediately after the microalgae were suspended in the nitrogen-free medium. Note that in all cases (${C}_{{x}_{0}}$ of 0.21, 0.52, 1.39 and 2.21 kg m^−3^), the reported decrease in chlorophyll content *W*_*chl*_ was greater during the first 4 days of starvation. Then the chlorophyll content *W*_*chl*_ continues to decrease but at a slower rate. This well-known result could be explained by the role of chlorophyll in the internal storage of nitrogen. Cells degrade their chlorophyll to support cell division and therefore maintain growth under conditions of nitrogen deprivation (Li et al. 2010; Van Vooren et al. 2012; Kandilian et al. 2014; Taleb et al. 2015, 2016; Scibilia et al. 2015).

Figure 1b shows the temporal evolution of the biomass concentration *C*_*x*_ of *H. pluvialis* grown in batch mode and subjected to sudden nitrogen starvation with initial biomass concentrations ${C}_{{x}_{0}}$ 0.21, 0.52, 1.39 and 2.21 kg m^−3^. The four batches reached biomass concentrations of 0.34, 1.59, 2.39 and 2.76 kg m^−3^ after 10 days of cultivation, respectively. This observation highlights the gradual increase in *C*_*x*_ over time when cultivated in a nitrogen-depleted medium. This behavior has been previously documented and discussed by Flynn et al. (1993) and Fan et al. (1998). Indeed, in nitrogen-deficient conditions, cells initially divide when nitrogen stress begins, but as the stress becomes more pronounced, cells eventually face mortality. It therefore depends on the ability of cells to continue dividing even in the absence of nitrogen.

While high light intensity has been observed to stimulate astaxanthin accumulation, our results revealed a noteworthy phenomenon occurring at very low initial biomass concentrations, specifically with ${C}_{{x}_{0}}$ of 0.21 kg m^−3^. Under these conditions, the growth rate substantially diminished, resulting in low biomass concentration and productivity. After 10 days of cultivation, *C*_*x*_, *P*_*x*_ and *S*_*x*_ were recorded as 0.34 kg m^−3^, 14.5 g m^−3^ d^−1^ and 0.43 g m^−2^ d^−1^, respectively.

**Fig. 2:**
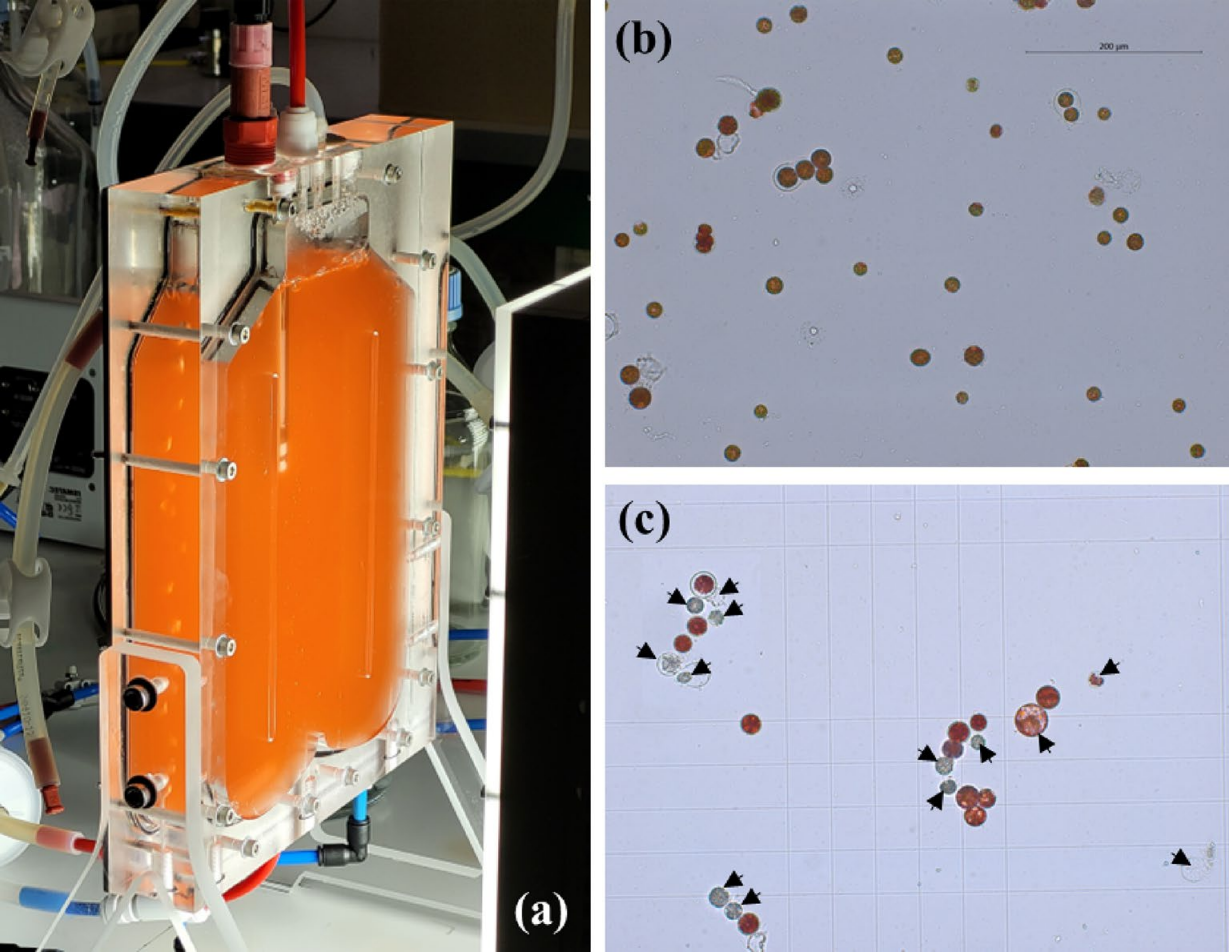
The 1-L airlift PBR (**a**) and the morphological changes (**b** and **c**) of H. pluvialis cells for an initial biomass concentration ${\mathrm{C}}_{{\mathrm{x}}_{0}}$ of 0.21 kg m^−3^ under nitrogen starvation and PFD of 250 µmol_hν_ m^−2^ s^−1^ at day 1 (**b**) and day 10 (**c**) of culture. The damaged or bleaching cells are indicated by arrows. Scale bar: 200 µm

Additionally, we observed cell bleaching and death (as seen in Fig. 2). A similar phenomenon had previously been noted by Wang et al. (2013a, b) when cultivating *H. pluvialis* in nitrogen starvation with a very low initial biomass concentration (${C}_{{x}_{0}}$= 0.1 kg m^−3^).

However, when the PBR was inoculated with higher biomass concentrations ${C}_{{x}_{0}}$(1.39 and 2.21 kg m^−3^), the final biomass concentrations reached 2.39 and 2.76 kg m^−3^, respectively, at the end of the culture. This corresponded to volumetric biomass productivities *P*_*x*_ of 99.66 and 55.33 g m^−3^ d^−1^, respectively. This observation aligns with previous findings by Van Vooren et al. (2012), Wang et al. (2013a, b), Kandilian et al. (2014) and Taleb et al. (2015), emphasizing the significant influence of initial biomass concentration on the resulting growth. Notably, the maximum volumetric biomass productivity *P*_*x*_ of 109 g m^−3^ d^−1^ was achieved with an initial biomass concentration ${C}_{{x}_{0}}$ equal to 0.52 kg m^−3^ (corresponding to an areal biomass productivity *S*_*x*_ of 3.27 g m^−2^ d^−1^). Although higher inoculation concentrations ${C}_{{x}_{0}}$ resulted in greater final biomass concentrations ${C}_{{x}_{f}}$, lower concentrations favored higher growth rates. Consequently, the growth rate was found to be most favorable under conditions with an initial biomass concentration ${C}_{{x}_{0}}$ of 0.52 kg m^−3^.

These differential growth patterns and pigment evolution over time can be elucidated by considering the role of light absorption within the culture. As previously demonstrated in the studies by Van Vooren et al. (2012) and Kandilian et al. (2014), the light transfer within microalgae cultures grown in PBRs under nitrogen-starvation conditions exerts a profound influence on both cell growth and metabolism. For cultures with low initial biomass concentrations, the lower cell density ensures enhanced accessibility of cells to the available light within the culture, thereby leading to heightened metabolic activity. A more comprehensive analysis of these results will be provided through the examination of the mean rate of photon absorption, as represented by MRPA in the subsequent sections.

### Effect on astaxanthin accumulation

**Fig. 3:**
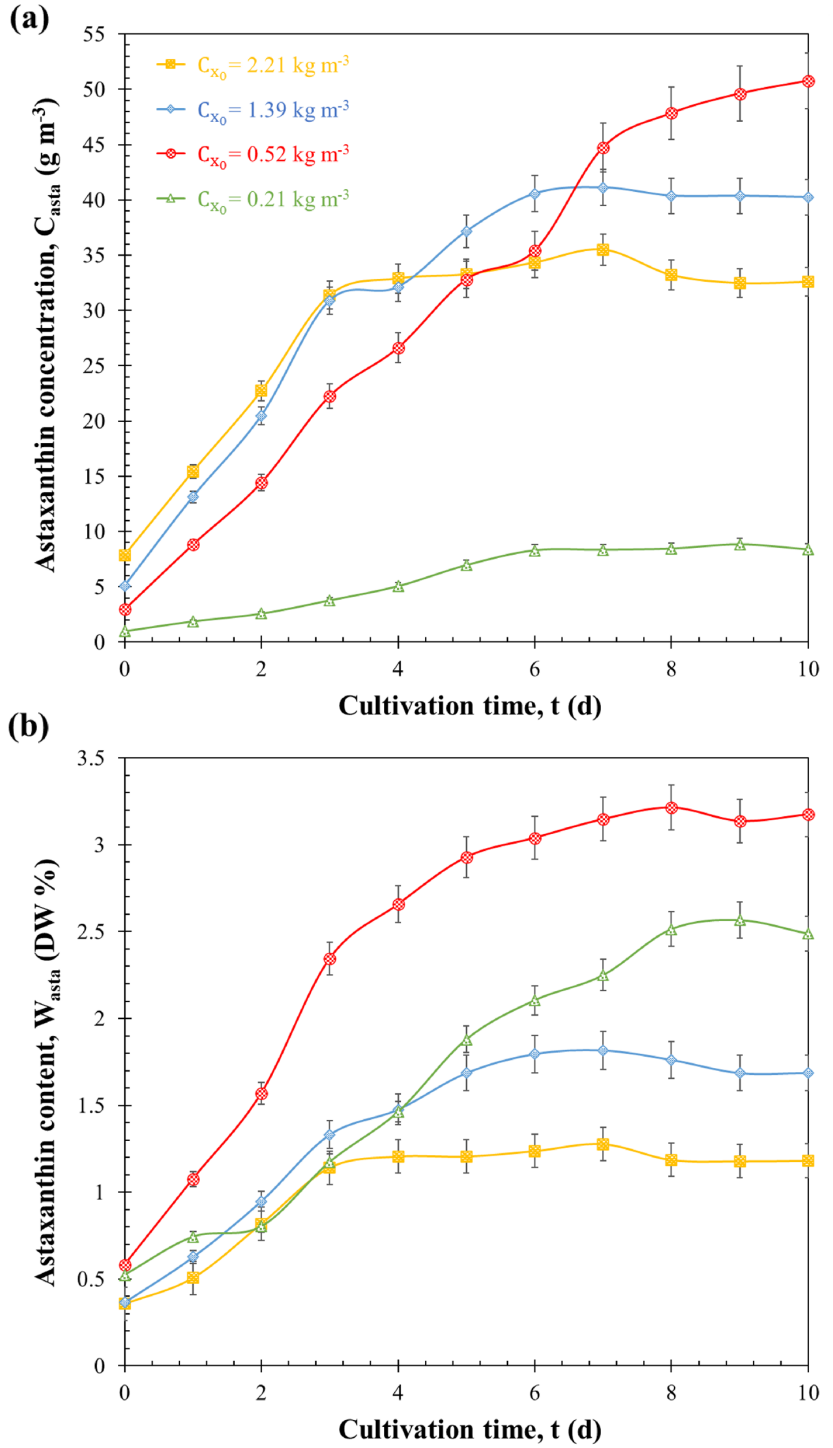
Astaxanthin concentration C_asta_ (**a**) and content W_asta_ (**b**) of H. pluvialis during sudden nitrogen starvation of batch cultures exposed to PFD of 250 µmol_hν_ m^−2^ s^−1^ with initial biomass concentrations ${\mathrm{C}}_{{\mathrm{x}}_{0}}$ equals to 0.21, 0.52, 1.39 and 2.21 kg m^−3^ as a function of cultivation time t. Data shown as mean ± SD, *n* = 3

Figure 3 shows the temporal evolution of the astaxanthin concentration *C*_*asta*_ and content *W*_*asta*_ for *H. pluvialis* grown in batch mode and subjected to sudden nitrogen starvation with initial biomass concentrations ${C}_{{x}_{0}}$ of 0.21, 0.52, 1.39 and 2.21 kg m^−3^. It indicates an immediate increase in astaxanthin concentration in cells following their suspension in nitrogen-free medium. Indeed, experiments with initial concentration ${C}_{{x}_{0}}$ of 0.21, 0.52, 1.39 and 2.21 kg m^−3^ featured cells that reached an astaxanthin concentration of 1.46, 2.66, 1.48 and 1.21% DW after 4 days, respectively. The culture with initial biomass concentration ${C}_{{x}_{0}}$ equal to 0.52 kg m^−3^ reached the maximum final astaxanthin concentration *C*_*asta*_ of 50 g m^−3^ and the maximum final astaxanthin content *W*_*asta*_ of 3.21% DW after 10 days of cultivation. This compared well with the astaxanthin content of 2.7 and 3.8% DW as reported by Wang et al. (2013a, b) for an optimal initial biomass concentration ${C}_{{x}_{0}}$ equal to 0.8 kg m^−3^ in the absence of nitrogen.

In the end, throughout the duration of the culture, it was observed that the ability of cells to accumulate astaxanthin exhibited greater strength under conditions of lower initial biomass concentration (${C}_{{x}_{0}}$= 0.52 kg m^−3^). Specifically, the astaxanthin content increased by approximately 90% in this condition, compared to an increase of only 70% when the initial biomass concentration was higher (${C}_{{x}_{0}}$= 2.21 kg m^−3^). Since all nitrogen starvation cultures were exposed to the same incident PFD (250 µmol_hν_ m^−2^ s^−1^), differences in light attenuation within the PBR and the cells’ radiative properties (which depend on pigment content) were the likely factors contributing to the observed variations in astaxanthin metabolism across different batches.

These findings suggest that a lower initial biomass concentration results in reduced light attenuation within the PBR. Consequently, this condition ensures that cells receive adequate photon absorption, which in turn activates carotenoid biosynthesis pathways, ultimately enhancing astaxanthin accumulation. It is worth noting that these results align with previous studies on the effect of initial biomass concentration (Wang et al. 2013a, b) and incident light intensity (Li et al. 2010; Zhang et al. 2018), all investigating astaxanthin accumulation by *H. pluvialis* cells under nitrogen-starvation conditions. These collective findings suggest that the improved astaxanthin accumulation is likely linked to better lighting conditions in the culture medium, impacting the transfer of light energy as a primary contributing factor.

### Effect on radiation characteristics of H. pluvialis

#### Mass absorption cross-section

**Fig. 4:**
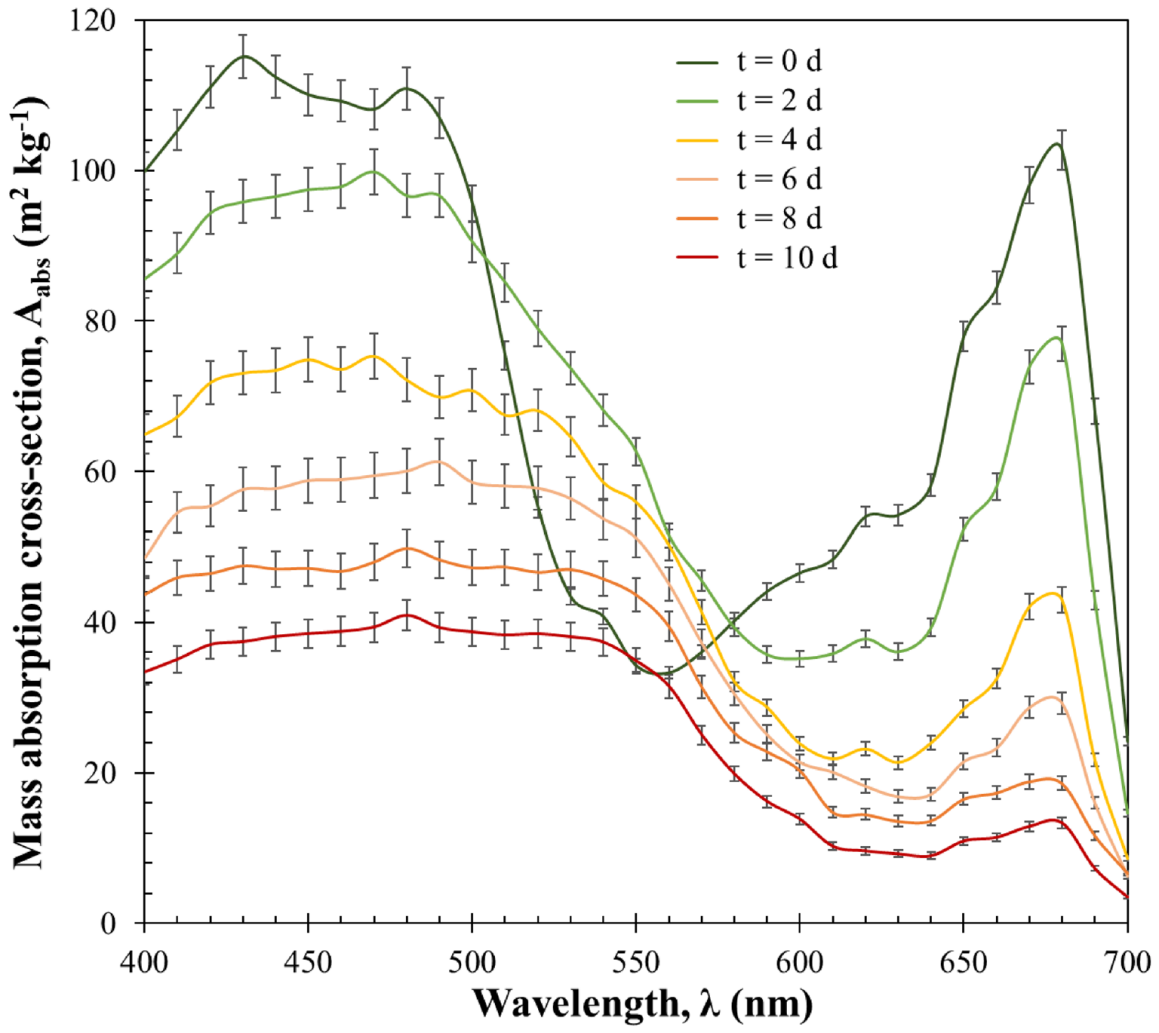
The spectral mass absorption cross-section ${\mathrm{A}}_{\mathrm{abs},\uplambda }$ of H. pluvialis during sudden nitrogen starvation of batch culture exposed to PFD of 250 µmol_hν_ m^−2^ s^−1^ with initial biomass concentration ${\mathrm{C}}_{{\mathrm{x}}_{0}}$ equal to 0.52 kg m^−3^ as a function of wavelength λ. Data shown as mean ± SD, *n* = 3

Figure 4 shows the temporal evolution of the measured spectral mass absorption cross-sections ${A}_{abs,\lambda }$ in the spectral region from 400 to 700 nm for *H. pluvialis* during sudden nitrogen starvation of the batch culture with an initial biomass concentration ${C}_{{x}_{0}}$ of 0.52 kg m^−3^.

Overall, ${A}_{abs,\lambda }$ exhibited a consistent decrease over time across all wavelengths within the PAR region. This trend was consistent even when different initial biomass concentrations were considered (data not shown). The overall decrease in ${A}_{abs,\lambda }$ over time was found mainly consistent with the continuous decrease in chlorophyll content *W*_*chl*_, as shown in Fig. 1a.

**Fig. 5:**
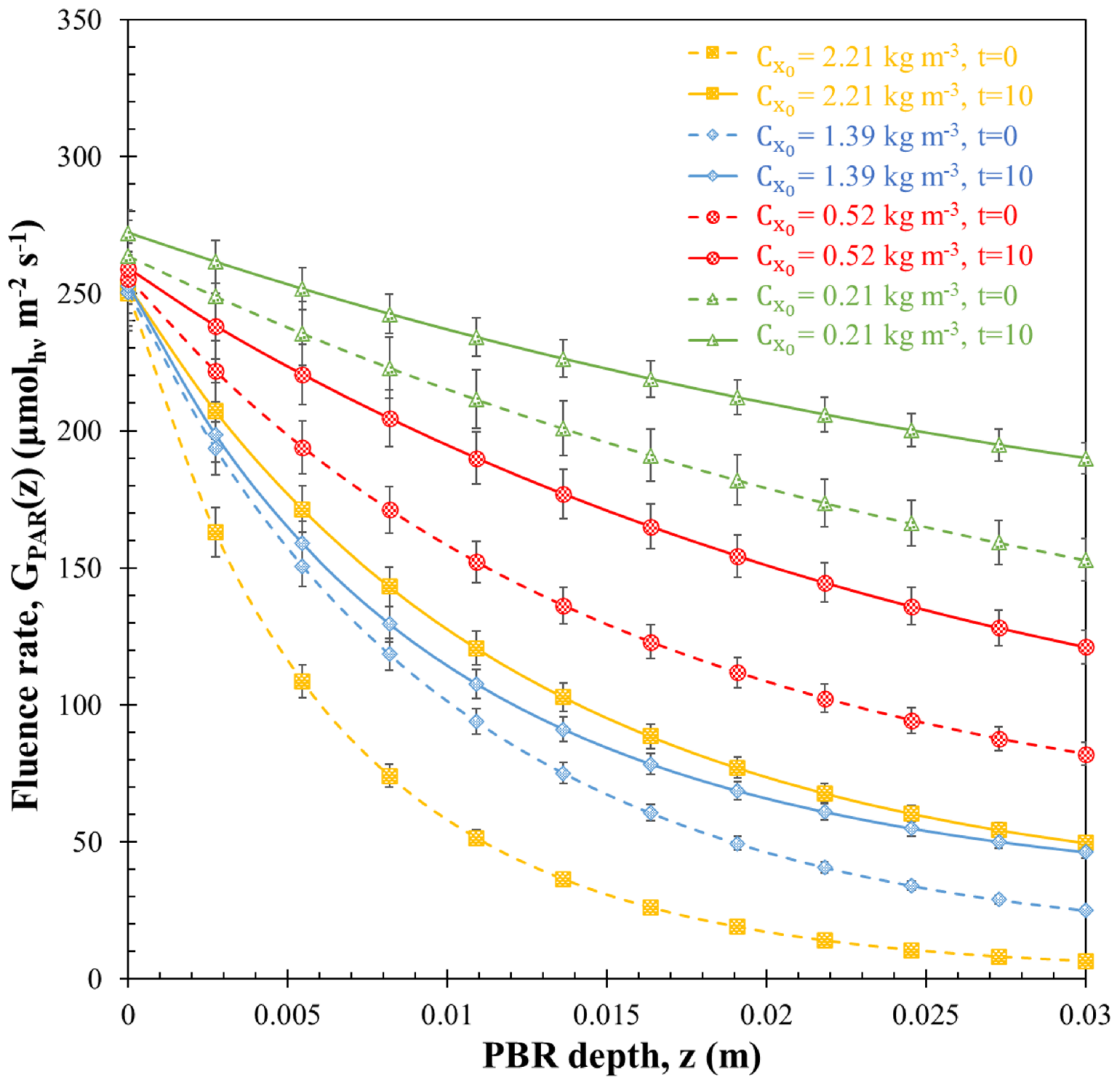
The PAR-averaged fluence rate $\mathrm{G}\left(\mathrm{z}\right)$ of H. pluvialis at the start (t = 0) and the end (t = 10) of sudden nitrogen starvation of batch cultures exposed to PFD of 250 µmol_hν_ m^−2^ s^−1^ with initial biomass concentrations ${\mathrm{C}}_{{\mathrm{x}}_{0}}$ equals to 0.21, 0.52, 1.39 and 2.21 kg m^−3^ as a function of PBR depth z. Data shown as mean ± SD, *n* = 3

The magnitude and the shape of the ${A}_{abs,\lambda }$ of *H. pluvialis* cultures changed slightly for cultures grown nitrogen starvation. For instance, the mass absorption cross-sections at 435 nm ${A}_{abs,435}$ and 676 nm ${A}_{abs,676}$, corresponding to chlorophyll a absorption peaks, decreased from their initial values of 115 and 102 m^2^ kg^−1^ to only 37 and 13 m^2^ kg^−1^, respectively, after 10 days for the culture with initial biomass concentration ${C}_{{x}_{0}}$ of 0.52 kg m^−3^. During the same time period, the chl-a content *W*_*chl-a*_ decreased from 0.8 to 0.06% DW, as later seen in the analysis of PAR-averaged fluence rate $G\left(z\right)$ within the PBR (Fig. 5).

It is important to note that changes in ${A}_{abs,\lambda }$ over time might also be influenced by alterations in cell size or the biochemical composition of the cells, such as pigments (Jonasz and Fournier 2007). Attributing these observed changes to any specific parameter is challenging due to the intricate biological response of *H. pluvialis* to nitrogen starvation, which involves significant transformations in morphological characteristics, pigment profiles, and content, as documented by prior research (Flynn et al. 1993; Fan et al. 1998).

#### Local fluence rate

Figure 5 shows the variation in the PAR-averaged fluence rate $G\left(z\right)$, in relation to the PBR depth *z*. This variation is based on the radiation characteristics observed during sudden starvation experiments, specifically at two time points: immediately (day 0) and after a 10-day period. These experiments were conducted across initial biomass concentrations ${C}_{{x}_{0}}$ of 0.21, 0.52, 1.39 and 2.21 kg m^−3^.

As expected, the fluence rate $G\left(z\right)$, at any given time, was larger for the cultures with smaller biomass concentration because of lower light attenuation. Interestingly, for all four batches, the fluence rate after 10 days of cultivation was larger than the initial fluence rate, despite the significant increase in biomass concentration (see Fig. 1b). This phenomenon can be attributed to the decrease in the mass absorption cross-section (as illustrated in Fig. 4), which had a more substantial impact on light transfer than the increase in biomass concentration. A similar outcome was observed during nitrogen starvation experiments with *Neochloris oleoabundans* and *Nannochloropsis oculata* in a flat-panel PBR, as reported by Pruvost et al. (2009) and Kandilian et al. (2014), respectively.

However, it is important to note that the fluence rate alone is not indicative of the amount of light absorbed by the cells (Pruvost and Cornet 2012). The absorption cross-section must also be taken into account, as it represents the photon-absorbing capacity, influenced by the pigmentation of the cells. This consideration becomes crucial in the context of nitrogen starvation, given the sharp decrease in absorption cross-section concurrent with an increase in available light, as indicated by the fluence rate. The dynamic changes in these values over time complicate the prediction of the overall impact on MRPA values, which combine both quantities, contributing to the effective light absorption by cells within the culture volume.

#### Mean rate of photon absorption

**Fig. 6:**
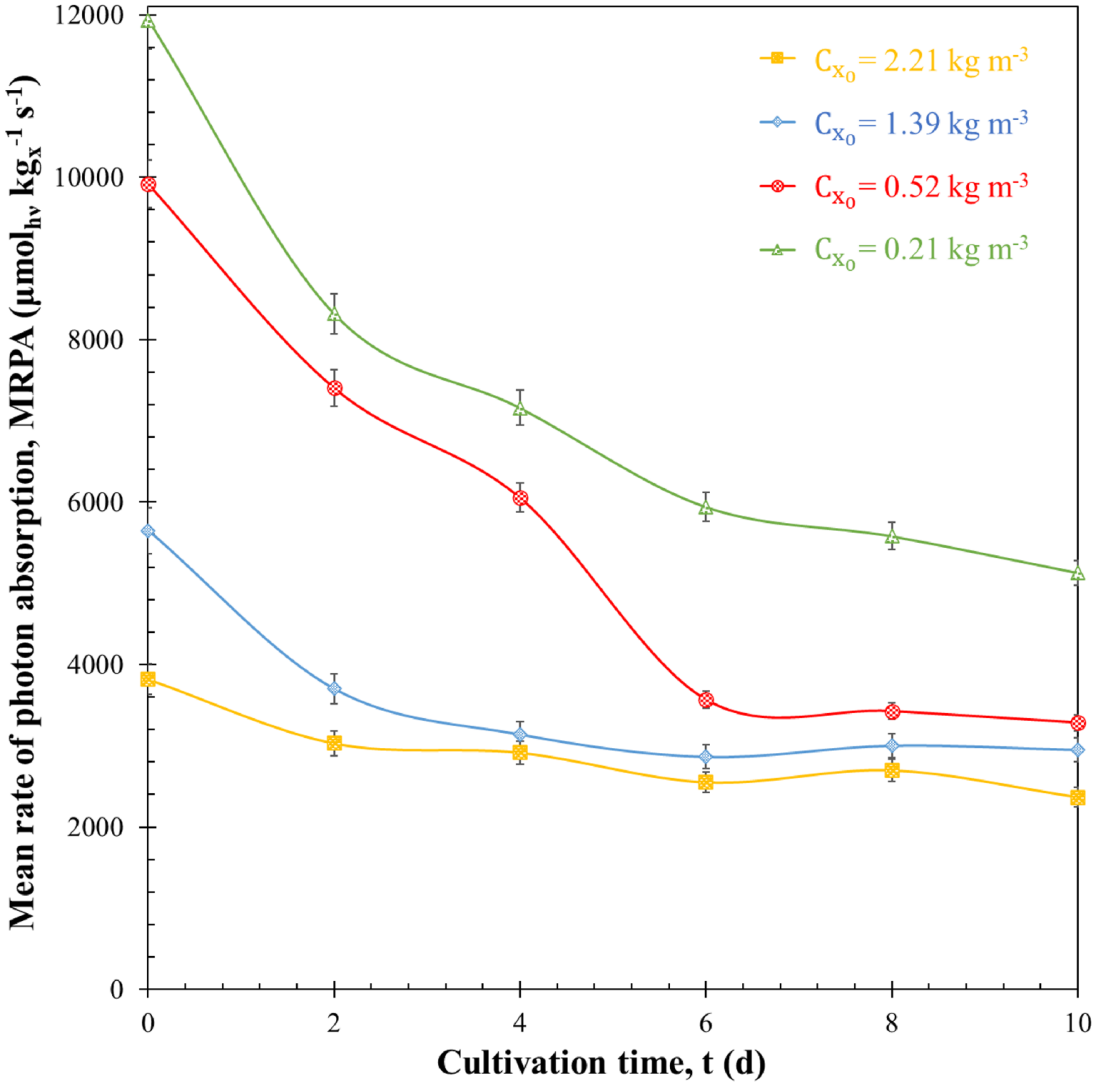
The mean rate of photon absorption MRPA of H. pluvialis during sudden nitrogen starvation of batch cultures exposed to PFD of 250 µmol_hν_ m^−2^ s^−1^ with initial biomass concentrations ${\mathrm{C}}_{{\mathrm{x}}_{0}}$ equals to 0.21, 0.52, 1.39 and 2.21 kg m^−3^ as a function of cultivation time t. Data shown as mean ± SD, *n* = 3

Figure 6 shows the mean rate of photon absorption MRPA as a function of time for each sudden nitrogen starvation cultivation. It was calculated using the corresponding experimentally measured daily average mass absorption cross-sections ${A}_{abs}$. Here also, the MRPA was larger for batches with smaller initial biomass concentration at all times. Considering the similar pigment content at inoculation, this could be attributed to the correspondingly larger fluence rate in the PBR (Fig. 5). In addition, MRPA decreased with time for all batches. For example, in the sudden nitrogen starvation experiment with ${C}_{{x}_{0}}$ of 0.52 kg m^−3^, the MRPA was 10000 µmol_hν_ kg_x_^−1^ s^−1^ initially but decreased to 3300 µmol_hν_ kg_x_^−1^ s^−1^ after 10 days. This may seem counterintuitive since the fluence rate increased during nitrogen starvation (Fig. 5). However, the decrease in the mass absorption cross-section dominated over the increase in the fluence rate. As previously suggested, the MRPA is indicative of the amount of photon absorbed by the microalgae, unlike the fluence rate $G\left(z\right)$ which ony represents the light available at a given position in the PBR. The decrease in MRPA demonstrates that, on average, the energy absorbed per cell decreased during nitrogen starvation. This may negatively impact both cells division and astaxanthin synthesis. Indeed, microalgae rely on the absorption of incident photon to carry out biochemical reactions. Their inability to absorb light could reduce their efficacy in performing photosynthesis and in fixating inorganic carbon (Williams and Laurens 2010).

### Astaxanthin productivity

Our previous examination of MRPA values has been expanded to assess their impact on astaxanthin metabolism in nitrogen-deficient cells under varying light attenuation conditions resulting from four different initial biomass concentrations. This trend, analogous to the observations made regarding TAG-lipids synthesis by Kandilian et al. (2014, 2019), suggests that if astaxanthin production is influenced by the rate of light absorption (termed “light stress”), then the rate of astaxanthin production should exhibit a correlation with MRPA. The daily average areal astaxanthin productivity ${\overline{S} }_{asta}$ and the daily average MRPA < $\overline{\mathcal{A} }$> were calculated at discrete time points using experimental data in accordance with Eq. 8 and Eq. 9.

**Fig. 7:**
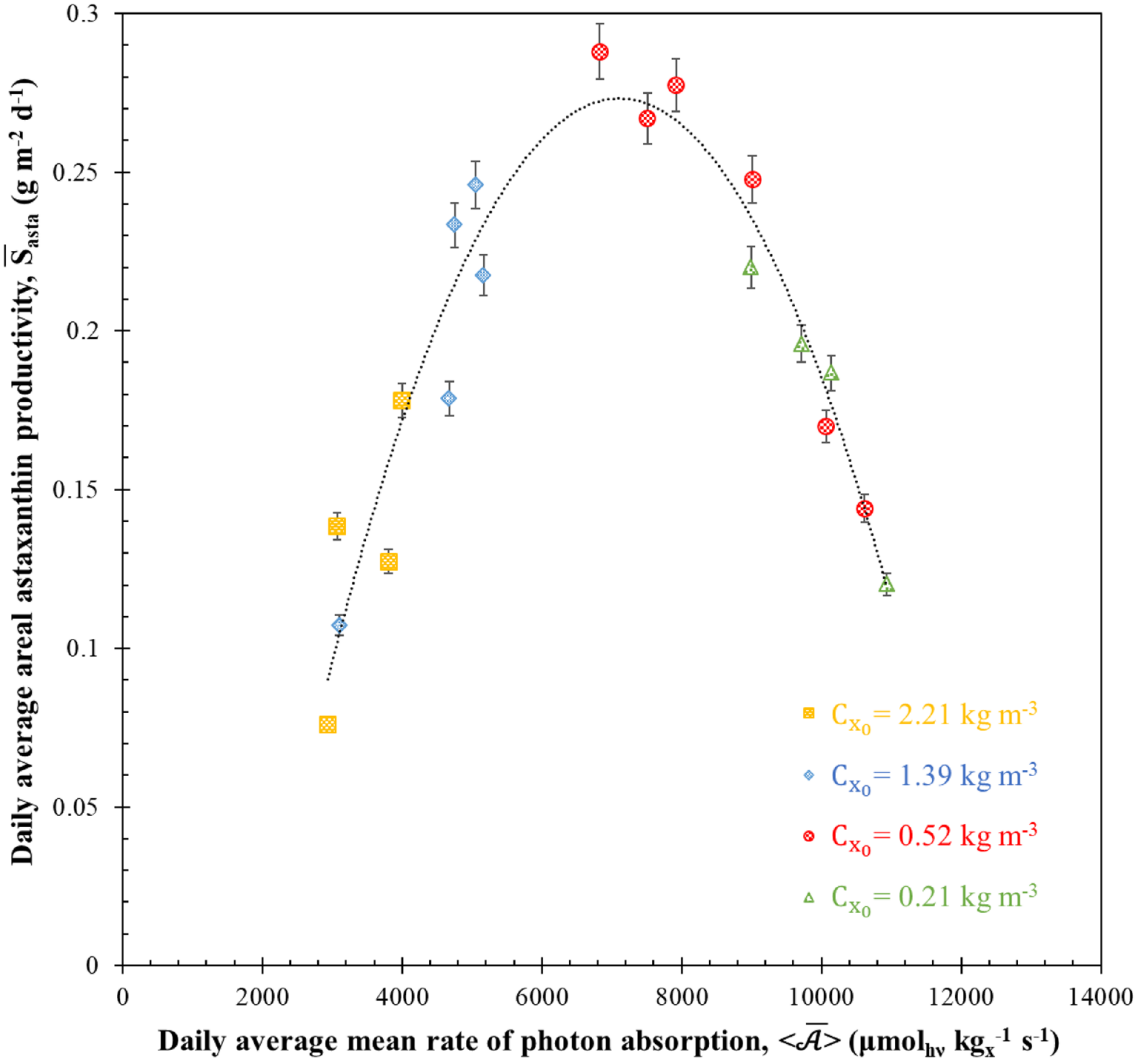
Evolution of daily average areal astaxanthin productivity ${\overline{\mathrm{S}} }_{\mathrm{asta}}$ of H. pluvialis during sudden nitrogen starvation of batch cultures exposed to PFD of 250 µmol_hν_ m^−2^ s^−1^ with initial biomass concentrations ${\mathrm{C}}_{{\mathrm{x}}_{0}}$ equals to 0.21, 0.52, 1.39 and 2.21 kg m^−3^ as a function of daily average mean rate of photon absorption < $\overline{\mathcal{A} }$>. Data shown as mean ± SD, *n* = 3

In Fig. 7, we present a graph depicting the relationship between the daily average areal astaxanthin production rate and the daily average MRPA for the sudden starvation experiments with four different initial biomass concentrations ${C}_{{x}_{0}}$. A notable parabolic relationship emerged, indicating a peak in ${\overline{S} }_{asta}$ at 0.29 ± 0.05 g m^−2^ d^−1^ when < $\overline{\mathcal{A} }$> reached 7000 ± 100 µmol_hν_ kg_x_^−1^ s^−1^ (all data are presented in Additional file 1). This relationship underscores that nitrogen starvation alone does not guarantee high astaxanthin production rates. It suggests that astaxanthin biosynthesis kinetics are also constrained by the photon absorption rate, represented by daily average MRPA < $\overline{\mathcal{A} }$>. This mirrors the situation of microalgae grown under optimal conditions, where biomass productivity is primarily limited by light availability.

Increasing MRPA per unit microalgae mass can be achieved by reducing biomass concentration. However, falling below the optimum MRPA value leads to decreased biomass productivity and incomplete light absorption in the PBR, as noted in prior studies (Pruvost and Cornet 2012; Takache et al. 2012). In such cases (known as kinetic regime), biomass productivity is limited by the microalgae’s biosynthesis rate.

Furthermore, increasing the daily average MRPA < $\overline{\mathcal{A} }$> beyond its optimal value results in decreased daily average astaxanthin productivity. However, due to reductions in chlorophyll content and mass absorption cross-section, complete light absorption in the PBR during nitrogen starvation becomes unattainable. This situation implies that the biological limit on astaxanthin accumulation in cells is reached. For example, increasing < $\overline{\mathcal{A} }$> from 3800 to 12000 µmol_hν_ kg_x_^−1^ s^−1^ on the first day of cultivation was achieved by reducing the initial biomass concentration ${C}_{{x}_{0}}$ from 2.21 to 0.21 kg m^−3^. Both experiments resulted in cells with 0.81% and 1.17% DW on days 2 and 3 of cultivation, respectively. However, the corresponding astaxanthin concentrations in the PBR differed significantly between the two batches (22.72 and 2.59 g m^−3^ at day 2 and 31.39 and 3.79 g m^−3^ at day 3, respectively). Thus, increasing MRPA did not affect the astaxanthin concentration per cell but led to smaller daily average astaxanthin productivity due to lower biomass concentration.

Additionally, there were differences in the temporal evolution of biomass concentration between experiments with initial biomass concentrations ${C}_{{x}_{0}}$ of 2.21–1.39 kg m^−3^ throughout the batch culture duration. However, both experiments had similar astaxanthin concentrations after 4 days of cultivation (*C*_*asta*_ = 32.51 g m^−3^). Interestingly, the daily average astaxanthin productivity of the PBR with an initial biomass concentration ${C}_{{x}_{0}}$ of 1.39 kg m^−3^ during the first 4 days of cultivation exceeded that of the PBR with an initial biomass concentration ${C}_{{x}_{0}}$ of 2.21 kg m^−3^ due to its lower initial astaxanthin concentration (*C*_*asta*_ = 5.08 g m^−3^). Nevertheless, between days 3 and 4, both experiments exhibited similar astaxanthin concentrations (see Fig. 3a) and therefore a similar daily average astaxanthin productivity (${\overline{S} }_{asta}$ = 0.041 ± 0.006 g m^−2^ d^−1^). Notably, they both maintained comparable values of daily average MRPA < $\overline{\mathcal{A} }$>. This is evident in Fig. 7, where data from both experiments clustered for daily average MRPA values < $\overline{\mathcal{A} }$> ranging from 3000 to 5000 µmol_hν_ kg_x_^−1^ s^−1^. This highlights the utility of our method for correlating MRPA with astaxanthin productivity in quantifying and optimizing “light stress”. Despite differences in biomass and chlorophyll concentrations, cultures with similar MRPA values demonstrated similar astaxanthin productivities.

In summary, these results suggest that optimizing astaxanthin productivity can be achieved in practice by adjusting the initial MRPA value, denoted as MRPA_0_, through changes in initial biomass concentration based on incident PFD and PBR thickness.

### Astaxanthin accumulation

**Fig. 8:**
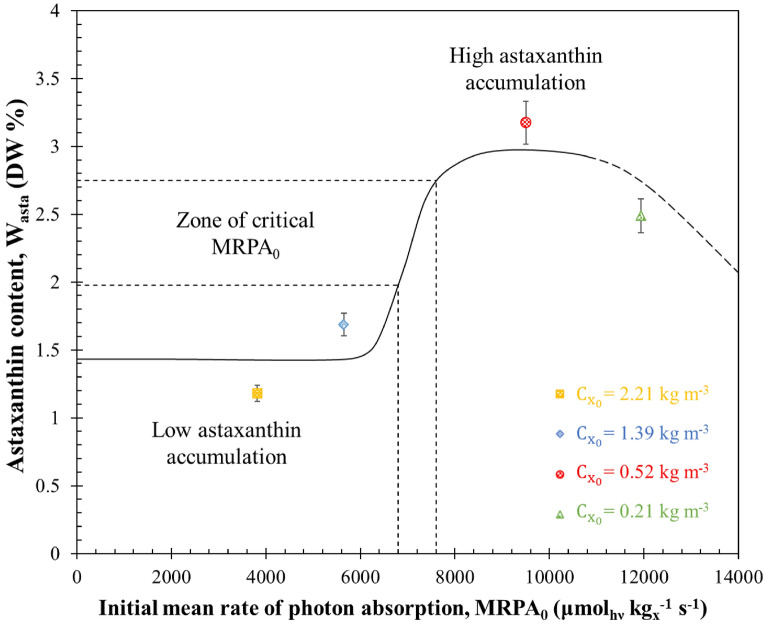
Cellular astaxanthin concentration W_asta_ after 10 days of sudden nitrogen starvation as a function of initial mean rate of photon absorption MRPA_0_. Data shown as mean ± SD, *n* = 3

The four experiments resulted in cultures with varying astaxanthin content *W*_*asta*_ after 10 days of sudden nitrogen starvation experiments, measuring 2.49%, 3.21%, 1.69%, and 1.18% DW for initial biomass concentrations ${C}_{{x}_{0}}$ of 0.21, 0.52, 1.39 and 2.21 kg m^−3^, respectively. These findings are depicted in Fig. 8, illustrating the relationship between astaxanthin cell content and the initial MRPA_0_. This figure highlights the presence of a critical initial MRPA value (${\mathrm{MRPA}}_{{0}_{\mathrm{cr}}}$) above which cells accumulate substantial quantities of astaxanthin.

Importantly, the estimated critical initial ${\mathrm{MRPA}}_{{0}_{\mathrm{cr}}}$ value is approximately 7000 ± 500 µmol_hν_ kg_x_^−1^ s^−1^. It’s worth noting that exceeding this critical ${\mathrm{MRPA}}_{{0}_{\mathrm{cr}}}$ value during the initial days of sudden starvation cultivation poses a significant challenge. This challenge arises from a notable decrease in MRPA observed during the initial cultivation days, as depicted in Fig. 6. This decline is a result of a rapid reduction in chlorophyll content (as shown in Fig. 1a) and an increase in biomass concentration (as shown in Fig. 1b). Consequently, achieving a substantial astaxanthin content in batch-cultured cells necessitates precise adjustment of the initial biomass concentration ${C}_{{x}_{0}}$ within the batch culture to attain MRPA_0_ values exceeding the critical ${\mathrm{MRPA}}_{{0}_{\mathrm{cr}}}$ of 7000 ± 500 µmol_hν_ kg_x_^−1^ s^−1^.

This critical ${\mathrm{MRPA}}_{{0}_{\mathrm{cr}}}$ value under nitrogen starvation corresponds to conditions where astaxanthin synthesis by cells outpaces the synthesis rates of carbohydrates, proteins, and other cellular components. Nitrogen starvation is known to be a potent stress condition for microalgae, impacting chlorophyll and protein biosynthesis. Microalgae exposed to nitrogen-deficient conditions tend to redirect their metabolic processes, resulting in the accumulation of lipids and carotenoids, as reported in prior studies (Berges et al. 1996; Cakmak et al. 2012; Schmollinger et al. 2014). In the case of *H. pluvialis*, the combination of nitrogen starvation and high light levels triggers starch degradation, the accumulation of carbohydrates and fatty acids, and an increased tricarboxylic acid cycle activity (Boussiba and Vonshak 1991; Recht et al. 2014). Astaxanthin synthesis is activated under nitrogen starvation to act as an electron sink, preventing excess free radical formation in the photosynthetic electron transport chain (Hu et al. 2008). Additionally, it has been shown that *H. pluvialis* enhances cyclic electron transport to protect the photosynthetic apparatus under nitrogen-starvation conditions (Scibilia et al. 2015).

It is important to highlight that prolonged exposure to high light conditions can disrupt the photosynthetic apparatus, leading to the activation of photoprotective mechanisms like non-photochemical quenching (NPQ) (Scibilia et al. 2015; Chekanov et al. 2019). NPQ serves to regulate and safeguard the photosynthetic apparatus when the absorption of light energy exceeds its utilization capacity in photosynthesis. In such cases, some absorbed light is dissipated as heat rather than being used for astaxanthin synthesis, potentially reducing astaxanthin productivity. This situation is reminiscent of the culture with an initial biomass concentration ${C}_{{x}_{0}}$ of 0.21 kg m^−3^, which exhibited the highest MRPA_0_ (12000 µmol_hν_ kg_x_^−1^ s^−1^) but had the lowest astaxanthin concentration (*C*_*asta*_ = 8.4 g m^−3^). As previously mentioned, this culture had minimal biomass concentration and productivity (*C*_*x*_ = 0.34 kg m^−3^ and *S*_*x*_ = 0.43 g m^−2^ d^−1^), along with a drastic reduction in chlorophyll content (*W*_*chl*_ = 0.05 ± 0.001% DW). This reduction led to a high rate of cell bleaching and mortality, as shown in Fig. 2. This phenomenon becomes more evident in Figs. 5 and 6, which reveal high fluence rates at various culture depths $G\left(L\right)$ and high mean rates of photon absorption MRPA during the 10-day batch cultivation. These observations confirm that, at each time, the incident light was not fully utilized by the cells for astaxanthin synthesis; instead, it dissipated as heat, ultimately reducing astaxanthin accumulation. These findings are consistent with results reported by Wang et al. (2013a, b) during *H. pluvialis* cultivation under nitrogen-starvation conditions with an extremely low initial biomass concentration (${C}_{{x}_{0}}$= 0.1 kg m^−3^).

In contrast, within the culture characterized by an initial biomass concentration ${C}_{{x}_{0}}$ of 0.52 kg m^−3^ and a MRPA_0_ value of approximately 9900 µmol_hν_ kg_x_^−1^ s^−1^, the daily average MRPA < $\overline{\mathcal{A} }$> (as illustrated in Fig. 7) played a pivotal role in ensuring a high rate of astaxanthin production. This favorable condition resulted in cells containing a substantial 3.21% of astaxanthin content in terms of dry weight (DW%). What’s particularly interesting in this scenario is the close alignment between the initial critical MRPA value, ${\mathrm{MRPA}}_{{0}_{\mathrm{cr}}}$, and the daily average MRPA < $\overline{\mathcal{A} }$>, which corresponds to the peak daily average areal astaxanthin productivity. This alignment can be attributed to a crucial observation: the most significant increase in astaxanthin concentration within cells and the peak daily productivity occurred during the initial 4 days of nitrogen starvation. During this critical timeframe, the daily average MRPA < $\overline{\mathcal{A} }$> closely mirrored the initial MRPA_0_, providing an explanation for the similarity in optimal values for both parameters.

Furthermore, it is worth mentioning that the calculation of areal productivities holds valuable insights when extrapolating the results to different culture systems. In the specific experiment with an initial biomass concentration ${C}_{{x}_{0}}$ set at 0.52 kg m^−3^, the highest achieved batch areal biomass productivity reached 3.27 g m^−2^ d^−1^, while astaxanthin productivity reached 0.29 g m^−2^ d^−1^. It’s important to note that our observations underline a significant potential for productivity enhancement through the optimization of MRPA within the PBR. This observation aligns with the findings of previous studies conducted by Pruvost and Cornet (2012) and Kandilian et al. (2014, 2019), where MRPA was effectively employed to optimize the biomass and lipid productivities of microalgae in PBRs. This emphasizes the versatility of MRPA as a valuable tool for optimizing astaxanthin productivity across a range of PBR scales. However, it’s crucial to underscore that the effectiveness of this optimization approach hinges on the rigorous conduct of a radiation transfer analysis to accurately estimate MRPA.

## Conclusion

The present study investigated four sudden starvation experiments with different initial biomass concentrations ${C}_{{x}_{0}}$ of 0.21, 0.52, 1.39 and 2.21 kg m^−3^, exposed all to an incident PFD of 250 µmol_hν_ m^−2^ s^−1^, to evaluate the influence of the light stress, as quantified by the light absorption rate represented by the mean rate of photon absorption MRPA on the astaxanthin production rate for *H. pluvialis*.

The results demonstrated the existence of a direct relation between the MRPA and the daily astaxanthin productivity of *H. pluvialis* cultures. They also indicated that astaxanthin synthesis in the PBR was physically limited by the MRPA. A maximum areal astaxanthin productivity of 0.29 ± 0.05 g m^−2^ d^−1^ was obtained, corresponding to MRPA equal to 7000 ± 100 µmol_hν_ kg_x_^−1^ s^−1^. In addition, a critical initial ${\mathrm{MRPA}}_{{0}_{\mathrm{cr}}}$ in excess of also 7000 ± 500 µmol_hν_ kg_x_^−1^ s^−1^ was required to trigger a large astaxanthin accumulation in *H. pluvialis* cells during nitrogen starvation up to 3.21% DW.

### Supplementary Information

**Additional file 1: Table S1.** Evolution of the daily average areal astaxanthin productivity ${\overline{S} }_{asta}$ and the daily average mean rate of photon absorption <$\overline{\mathcal{A} }$> of Haematococcus pluvialis cells during sudden nitrogen starvation of batch cultures exposed to PFD of 250 µmol_hν_ m^-2^ s^-1^ with initial biomass concentrations ${C}_{{x}_{0}}$equals to 0.21, 0.52, 1.39 and 2.21 kg m^-3^.

## Data Availability

Main data generated or analyzed during this study are included in this published article (and its supplementary information files).

## Nomenclature

< $\mathcal{A}$>: Mean rate of photon absorption [µmol_hν_ kg_x_^−^^1^ s^−^^1^]
< $\overline{\mathcal{A} }$>: Daily average mean rate of photon absorption [µmol_hν_ kg_x_^−^^1^ s^−^^1^]
${\mathcal{A}}_{\uplambda }\left(\mathrm{z}\right)$: Spectral local rate of photon absorption [µmol_hν_ kg_x_^−^^1^ s^−^^1^]
${\mathrm{A}}_{\mathrm{abs},\uplambda }$: Spectral mass absorption cross-section [m^2^ kg^−^^1^]
C_asta_: Astaxanthin concentration [g m^−^^3^]
C_chl-a_: Chlorophyll a concentration [g m^−^^3^]
C_chl-b_: Chlorophyll b concentration [g m^−^^3^]
C_x_: Biomass concentration [kg m^−^^3^]
${\mathrm{G}}_{\uplambda }(z)$: Spectral fluence rate [µmol_hν_ m^−^^2^ s^−^^1^]
$\mathrm{G}\left(\mathrm{z}\right)$: PAR-averaged fluence rate [µmol_hν_ m^−^^2^ s^−^^1^]
L: Thickness of the PBR [m]
PFD: Photon flux density [µmol_hν_ m^−^^2^ s^−^^1^]
P_x_: Volumetric biomass concentration [g m^−^^3^ d^−^^1^]
${\overline{\mathrm{S}} }_{\mathrm{asta}}$: Daily average areal astaxanthin productivity [g m^−^^2^ d^−^^1^]
S_light_: Illuminated area of the PBR [m^2^]
S_x_: Areal biomass productivity [g m^−^^2^ d^−^^1^]
V_r_: Working volume of the PBR [m^3^]
W_asta_: Astaxanthin content [DW %]
W_chl_: Chlorophyll content [DW %]
z: Culture depth in the PBR [m]
λ: Light wavelength [nm]

## Abbreviations

BBM: Bold’s Basal Medium
chl-a: Chlorophyll a
chl-b: Chlorophyll b
DMSO: Dimethyl sulfoxide
DW: Dry weight
LED: Light emitting diode
LRPA: Local rate of photon absorption
MRPA: Mean rate of photon absorption
OD: Optical density
PAR: Photosynthetically active region
PBR: Photobioreactor
PBS: Phosphate buffer saline
TAG: Triacylglycerol

## Index

0: Initial
asta: Astaxanthin
cr: Critical
t: Time
x: Biomass
