# Supplementary material for: Influence of light absorption rate on the astaxanthin production by the microalga Haematococcus pluvialis during nitrogen starvation
Source: Bioresour Bioprocess. 2023 Nov 9;10(1):78. doi: 10.1186/s40643-023-00700-0 (PMC10992552; doi:10.1186/s40643-023-00700-0)
Supplement: Supplementary file 1 — Additional file 1: Table S1. Evolution of the daily average areal astaxanthin productivity ${\overline{S} }_{asta}$S¯asta and the daily average mean rate of photon absorption <$\overline{\mathcal{A} }$A¯> of Haematococcus pluvialis cells during sudden nitrogen starvation of batch cultures exposed to PFD of 250 µmolhν m-2 s-1 with initial biomass concentrations ${C}_{{x}_{0}}$Cx0equals to 0.21, 0.52, 1.39 and 2.21 kg m-3. [file 40643_2023_700_MOESM1_ESM.docx]

# Additional file: Influence of light absorption rate on the astaxanthin production by the microalga Haematococcus pluvialis during nitrogen starvation

Khadija SAMHAT^1,2,*^, Antoinette KAZBAR^3,4^, Hosni TAKACHE^5^, Ali ISMAIL^2^, Jeremy PRUVOST^1^

^1^ Nantes University, Oniris, CNRS, GEPEA, UMR 6144, F-44600 Saint-Nazaire, France

^2^ Lebanese University, Platform for Research and Analysis in Environmental Sciences, Doctoral School of Science and Technology, Rafic Hariri Campus, Beirut, Lebanon

^3^ Algosource, 7 Rue Eugène Cornet, F-44600 Saint-Nazaire, France

^4^ Bioprocess Engineering, Wageningen University and Research, Wageningen, Netherlands

^5^ Bio-Information Research Laboratory (BIRL), The Higher Institute of Biotechnologies of Paris (Sup’biotech), 66 rue Guy Môquet, 94800 Villejuif, France

^*^corresponding author: [k.samhat.95@gmail.com](mailto:k.samhat.95@gmail.comr)

Table 1: Evolution of the daily average areal astaxanthin productivity $\bar{S}_{asta}$ and the daily average mean rate of photon absorption <$\bar{\mathcal{A}}$> of Haematococcus pluvialis cells during sudden nitrogen starvation of batch cultures exposed to PFD of 250 µmol_hν_ m^-2^ s^-1^ with initial biomass concentrations $C_{x_{0}}$equals to 0.21, 0.52, 1.39 and 2.21 kg m^-3^.

| ***Initial biomass concentration***  $\boldsymbol{C}_{\boldsymbol{x}_{\boldsymbol{0}}}$***(kg m^-3^)*** | ***Daily average areal astaxanthin productivity***  ${\bar{\boldsymbol{S}}}_{\boldsymbol{asta}}$ ***(g m^-2^ d^-1^)*** | ***Daily average MRPA***  ***<***$\bar{\mathcal{A}}$***> (µmol_hν_ kg_x_^-1^ s^-1^)*** |
| --- | --- | --- |
| **2.21** | 0.14 ± 0.01 | 3072 |
|  | 0.08 ± 0.02 | 2935 |
|  | 0.18 ± 0.03 | 4001 |
|  | 0.13 ± 0.02 | 3806 |
| **1.39** | 0.22 ± 0.02 | 5164 |
|  | 0.25 ± 0.02 | 5051 |
|  | 0.18 ± 0.01 | 4677 |
|  | 0.23 ± 0.02 | 4754 |
|  | 0.11 ± 0.01 | 3103 |
| **0.52** | 0.14 ± 0.02 | 10617 |
|  | 0.25 ± 0.03 | 9011 |
|  | 0.29 ± 0.05 | 6850 |
|  | 0.28 ± 0.05 | 7921 |
|  | 0.27 ± 0.04 | 7512 |
|  | 0.17 ± 0.02 | 10066 |
| **0.21** | 0.12 ± 0.01 | 10937 |
|  | 0.19 ± 0.01 | 10141 |
|  | 0.20 ± 0.03 | 9714 |
|  | 0.22 ± 0.03 | 8994 |
